# Is Natural Orifice Specimen Extraction Surgery Really Safe in Radical Surgery for Colorectal Cancer?

**DOI:** 10.3389/fendo.2022.837902

**Published:** 2022-02-21

**Authors:** Gang Liu, Lianghui Shi, Zehui Wu

**Affiliations:** ^1^ Department of Gastrointestinal Surgery, The First Affiliated Hospital of Wannan Medical College, Wuhu, China; ^2^ Department of General Surgery, The First Affiliated Hospital of Anhui Medical University, Hefei, China

**Keywords:** colorectal cancer, natural orifice specimen extraction, transanal, postoperative, early safety

## Abstract

**Background:**

The main feature of *natural orifice specimen extraction (NOSE)* is its avoidance of an auxiliary abdominal incision. The safety of NOSE remains controversial. This study aimed to investigate the early safety of transanal NOSE in the treatment of sigmoid colon and upper rectal cancer from the follow aspects: clinical and pathological characteristics, inflammatory and immune indicators and postoperative complications.

**Methods:**

Data from 125 patients diagnosed with sigmoid colon, and upper rectal cancer by gastrointestinal surgery in the First Affiliated Hospital of Wannan Medical College from January 2017 to June 2020 were analyzed. Patients were assigned to two surgical groups: *Conventional laparoscopic-assisted radical resection for CRC* (*CLA*, 75cases) and *laparoscopic-assisted radical resection for CRC with NOSE* (*La-NOSE*, 50 cases). The following were compared: clinical and pathological characteristics; intraoperative, bacteriological, and oncological results; postoperative inflammation and immune response indexes. Bacteriological results were obtained by aerobic and anaerobic bacterial culture of peritoneal wash fluid and oncology results by cytological analysis of peritoneal wash liquid exudation. Inflammation indicators included postoperative C-reactive protein (CRP) and procalcitonin (PCT) trend reactions. The immune index was the level of postoperative T lymphocytes (CD3, CD4/CD8). All data were analyzed by using SPSS statistical version 18.0 for windows. Measurement data are presented as the means ± standard deviations, and two-group comparisons were performed using the t-test. Comparisons of count data were performed using the chi-square test. *p <0.05* indicates that the difference was statistically significant.

**Results:**

The bacterial culture positive rate was not significant in the La-NOSE group (15/50 vs 19/75) than in the CLA group. The *exfoliative cytology (EC)* rate of the peritoneal wash fluid was 0 in both groups.The La-NOSE group had a significantly higher postoperative day 2(POD2) CRP and PCT level than the CLA group. The POD2 CD3 and CD4/CD8 levels were higher in the La-NOSE group than in the CLA group. There was no significant difference in the incidence of postoperative complications between the two groups (La-NOSE group vs CLA group: 3/50 vs 6/75) (*p>0.05*).

**Conclusions:**

Although the incidence of intra-abdominal contamination is high, it does not develop into a severe infectious disease, and does not lead to the implantation of free tumor cells into the abdominal cavity. Therefore, it is safe for the NOSE to treat colorectal cancer.

## Background

Colorectal cancer (CRC) is a common malignant tumor. The global malignant tumor statistics for 2018 show that CRC morbidity ranks fourth among cancers, accounting for 10% of all malignant tumors, and that CRC mortality ranks second, accounting for 9% of cancer deaths (1). The main treatment for CRC is still surgical resection. A large number of studies have confirmed that compared with open surgery, laparoscopic-assisted colorectal cancer surgery has obvious advantages in short-term efficacy, and there is no significant difference in long-term efficacy (2–6).

At present, laparoscopic-assisted CRC surgery has been widely used in clinical practice and is extremely important in the field of CRC surgery. However, laparoscopic-assisted mini-laparotomy for CRC requires a micro-incision of approximately 5 cm in the abdomen during the removal of colorectal specimens and the reconstruction of the digestive tract. With the development of technology and concepts, natural orifice specimen extraction (NOSE) surgery has been proposed and applied to the clinical treatment of CRC. It avoids abdominal auxiliary incisions and has better short-term efficacy, including a quick postoperative recovery time, relief of postoperative pain, improved cosmetic outcomes, and a low rate of postoperative incision-related complications (incisional hernia and incision infection), which are welcomed by most surgeons (7–12).

Despite the many advantages of the NOSE operation, this method requires opening the intestine in the abdominal cavity; removing the specimen through the distal intestinal cavity, as well as gastrointestinal reconstruction and other specific procedures, which may increase the ectopic intestinal bacteria in the abdominal cavity. Contamination and squeezing of tumor specimens when taking specimens may increase the risk of ectopic implantation of tumor cells into the abdominal cavity. Therefore, this study aimed to explore the safety of transanal-NOSE CRC surgery, especially in terms of ectopic bacterial contamination of the abdominal cavity, and ectopic tumor implantation.

## Methods

This was a retrospective analysis of clinical data of patients diagnosed with sigmoid colon, and upper rectal cancer by gastrointestinal surgery from the gastrointestinal surgery of the First Affiliated Hospital of Wannan Medical College from January 2017 to June 2020. Inclusion criteria: (1) age: 18-75 years old; (2) preoperative pathological diagnosis with sigmoid nodules, and upper rectal cancer, (3) the largest tumor diameter <5 cm; (4) BMI ≤ 28 kg/m^2^; (5) preoperative imaging(CT and MRI, 2-3days before operation) stages of T1, T2 and T3. The exclusion criteria were as follows: (1) other primary tumors; (2) metastasis to other organs or extensive implantation and metastasis of the abdominal cavity; (3) previous bowel surgery due to bowel disease; (4) severe liver or kidney disease; or (5) neoadjuvant therapy or targeted therapy before surgery. According to the above criteria, a total of 125 patients were enrolled from January 2017 to June 2020. Patients were randomly assigned to receive one of two operation methods and divided into the La-NOSE group and CLA group according to the operation method: there were 50 cases in the La-NOSE group and 75 cases in the CLA group. The two groups of patients were matched according to clinical and pathological characteristics, including age, sex, American Society of Anaesthesiologists (ASA) classification, BMI, tumor size, tumor location, preoperative T stage, and tumor differentiation. For the sigmoid colon or middle and high rectum, the following surgical techniques were used.

### Perioperative Management

All patients underwent surgical treatment without conversion to open laparotomy. All operations were performed by the same team, and the surgeon had 10 years of laparoscopic surgery experience. The perioperative diagnosis and treatment plan strictly adhered to the guidelines of the National Comprehensive Cancer Network (NCCN), and all patients had a preoperative treatment plan completed by the same team. Before the operation, various laboratory and imaging examinations, colonoscopy, chest computed tomography scan or X-ray, abdominal computed tomography scan or pelvic magnetic resonance imaging were completed. The day before surgery, oral polyethylene glycol electrolyte powder catharsis was given, and a large dose (2.0 L) of soapy water was used for the enema on the morning of the operation. After the operation, intravenous self-controlled analgesia, standardized pain management, and opioid or NSAID analgesic drugs were added if necessary. In addition, according to the team’s clinical experience, all patients were given oral quinolones and nitroimidazole derivatives combined with oral preventive anti-infection on the second day after admission, and the catheter was removed on the third day after surgery.

### NOSE Surgical Operation

For the NOSE technique, the simple method was to use the five-hole method to create a 12 mmHg pneumoperitoneum. Upon completion of colorectal dissociation, mesenteric vascular ligation and lymph node dissection were performed. Rectal cancer separates the rectum under laparoscopy to less than 5 cm distal to the tumor, and separates the rectum by approximately 3 cm below the tumor (the sigmoid colon cancer separates the rectum from the peritoneum and separates the rectum from the tumor by approximately 5 cm below the tumor). Here, iodophor solution was sufficient. After rinsing the end of the rectum, the end of the rectum was cut, and the rectum was closed. The disposable sterile protective sleeve was placed into the abdominal cavity with a Trocar, the oval forceps were inserted through the anus, one end of the specimen bag was clamped, and the bag was led out from the anus, leaving the other end of the specimen bag in the abdominal cavity. The anvil was inserted into the abdominal cavity through the specimen bag from the anus. The proximal colon was closed to make an incision of approximately 2 cm in the mesentery. The circular anvil of the stapler was inserted through the incision into the proximal end of the colon, the incision was closed with a cut. The device was broken, and then, the central rod of the nail holder was poked out through the small mouth. The anus was enlarged, the oval forceps were placed through the specimen bag, and the specimen was pulled out. Then the end of the rectum was closed with a closure device. The main body of the stapler was inserted through the anal and rectal channels, and the central rod was drawn through the closed end of the rectum to pierce the anastomosis. After docking with the proximal end, the anastomosis was triggered. The abdominal cavity was repeatedly flushed with sterile saline to check whether the anastomosis was bleeding, and the card was punched through the right lower abdomen. The hole was placed, and the drainage tube is fixed.

### Statistical Methods

All data were analyzed by using SPSS statistical version 18.0 for windows (IBM Crop, Armonk, NY, United States). Measurement data are presented as the means ± standard deviations, and two-group comparisons were performed using the t-test. Comparisons of count data were performed using the chi-square test or Fisher’s exact test. *p <0.05* indicates that the difference was statistically significant.

## Results

### Clinical and Pathological Characteristics

From January 2017 to June 2020, a total of 125 patients were enrolled, including 50 in the La-NOSE group and 75 in the CLA group. The clinical and pathological data of the patients are shown in **Table 1**. There were no significant differences in age, sex, BMI, ASA score, tumor size, tumor location, preoperative T stage, and tumor differentiation between the La-NOSE group and the CLA group (**Table 1**).

**Table 1:** Clinical and pathological characteristics.

Features and results	La-NOSE Group (n = 50)	CLA Group (n = 75)	*P-*value
Sex			
Female	21	30	0.824
Male	29	45	
Age (years, mean ± SD)	60.68 ± 9.90	58.00 ± 10.11	0.146
BMI (kg/m^2^, mean ± SD)	23.35 ± 4.11	23.86 ± 3.82	0.480
ASA Class			
I-II	44	62	0.416
III-IV	6	13	
Tumor size (cm, mean ± SD)	3.29 ± 1.00	3.53 ± 1.01	0.209
Tumor location			
Sigmoid colon	23	34	0.942
Upper rectum	27	41	
Preoperative T stage			
T1	12	17	0.481
T2	25	31	
T3	13	27	
Degree of differentiation			
Highly differentiated	12	19	0.957
Medium differentiation	22	31	
Poorly differentiated	16	25	

The intraoperative results are shown in **Table 2**. There were no significant differences in total operation time (145.56 min vs 142.11 min), the estimated intraoperative blood loss (63.94 ml vs 62.55 ml) and the number of lymph nodes dissected (19.58 vs 18.99) were not significantly different between the two groups (**Table 2**).

**Table 2:** Intraoperative and postoperative results.

Results	La-NOSE Group (n = 50)	CLA Group (n = 75)	*P*-value
Operation time [min, mean ± SD]	145.56 ± 11.37	142.11 ± 12.83	0.126
intraoperative blood loss [ml, mean ± SD]	63.94 ± 27.76	62.55 ± 26.99	0.780
Lymph node harvest [pieces, mean ± SD]	19.58 ± 2.11	18.99 ± 1.98	0.112
EC-test results			
+	0	0	–
–	50	75	
Bacterial culture results			
+	15	19	0.566
–	35	56	
Postoperative complications	3	6	0.944
Incisional infection	0	2	0.516
Anastomotic leakage	1	1	1.000
Anastomotic stricture obstruction	0	1	1.000
Defecation incontinence	1	0	0.400
lung infection	1	2	1.000

### Laboratory Testing Results

Bacteriological results are shown in **Table 2**. In the La-NOSE group, 15 cases of peritoneal washing liquid bacterial culture were positive, and all were positive for Escherichia coli, with a positive rate of 30.00%(15/50). In the CLA group, 19 cases of liquid bacterial cultures of peritoneal washes were positive, all with E. coli, with a positive rate of 25.33%(19/75). The difference between the two groups was not significant.

**Figure 1:**
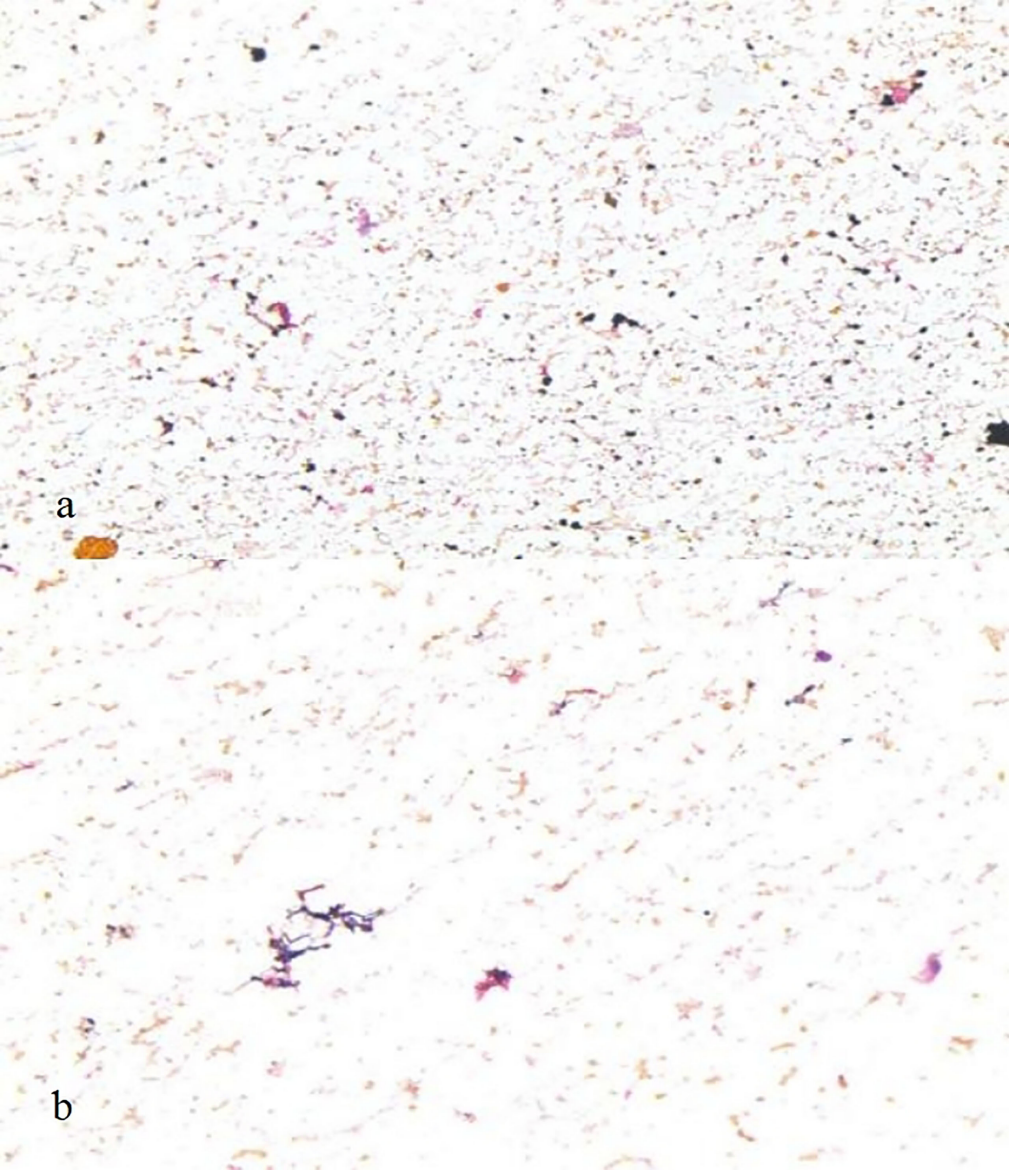
Laparoscopic wash fluid exfoliation cytology (EC) test after La-NOSE and CLA surgery: a large number of mesothelial cells and lymphocytes are evident by microscopy. a, La-NOSE group; b, CLA group.

The EC-test results are shown in **Table 2** and **Figure 1**. In both groups, no tumor cells were found in the cytological examinations of the peritoneal washes.

 The inflammatory and immune indicators are shown in (**Table 3**). The postoperative day 2 (POD2) CRP level (98.76 mg/L vs 78.54 mg/L, p=0.003) in the La-NOSE group was higher than that in the CLA group and the difference was statistically significant. Compared with that of the CLA group, the CPT level of the La-NOSE group was also statistically significantly different. Compared with those in the CLA group, POD2 CD3 (66.79% vs 63.14%, p=0.041), and the POD7 was also of significantly difference. POD2 CD4/CD8 (1.37 vs 1.21, p=0.001) in the La-NOSE group were increased, and the differences were statistically significant different.

**Table 3:** The inflammatory and immune indicators results.

INDICATORS		La-NOSES	CLA	*p*
CRP (mg/L)	BOD	3.41 ± 1.38	3.47 ± 1.78	0.806
	POD2	98.76 ± 33.61	78.54 ± 39.24	0.003
	POD7	15.12 ± 5.20	14.09 ± 5.30	0.287
PCT (ng/ml)	BOD	0.217 ± 0.11	0.24 ± 0.11	0.304
	POD2	4.02 ± 1.45	3.30 ± 1.42	0.007
	POD7	0.266 ± 0.15	0.30 ± 0.14	0.265
CD3 (%)	BOD	76.47 ± 6.02	75.33 ± 9.63	0.416
	POD2	66.79 ± 10.37	63.14 ± 9.16	0.041
	POD7	73.45 ± 7.71	69.23 ± 11.91	0.017
CD4/CD8	BOD	1.67 ± 0.19	1.69 ± 0.30	0.659
	POD2	1.37 ± 0.28	1.21 ± 0.26	0.001
	POD7	1.56 ± 0.24	1.61 ± 0.21	0.298

BOD, before operative day; POD2, the postoperative day 2; POD7, the postoperative day 7.

### Postoperative Complications

The incidence of postoperative complications is shown in **Table 2**. The postoperative complication rate was 6.00%(3/50) in the La-NOSE group; The complication rate was 8.00%(6/75) in the CLA group. The difference in the incidence of postoperative complications between the two groups was not statistically significant. There was one case of anastomotic leakage in each group, and anastomotic healing occurred after fasting and circulatory washing. No secondary surgery was performed (**Table 2**).

## Discussion

NOSE is an innovative technology in the field of laparoscopic surgery that is favored by an increasing number of young doctors. According to whether the La-NOSE procedure met the criteria for sterility, this study enrolled 50 patients undergoing La-NOSE peritoneal washing liquid for bacterial culture, among whom 15 cases were positive by bacterial culture, and the positive rate was 30.00%(15/50), with all positive for E. coli. None of the patients had clinical manifestations of abdominal infection until discharge. In the La-NOSE group, An elderly diabetic presented with yellow stool on the fifth day after surgery. Fasting and circular abdominal irrigation, anastomotic healing. The occurrence of Anastomotic leakage may be related to the patient’s arteriosclerosis and insufficient blood supply to the anastomotic site; One patient had postoperative fecal incontinence, was discharged after treatment, and had no anal dysfunction after three months of follow-up. The second operation may be due to the patient’s vascular sclerosis and reduced blood supply to the anastomosis. Of the 75 patients in the CLA group, 19 were positive by bacterial culture, all with E. coli, with a positive rate of 25.33%(19/75); Among them, Postoperative infection of abdominal auxiliary incision occurred in 2 cases, and the incision healed after repeated debridement and dressing changes; Another patient had fecal drainage on the drainage bag on the 4th day after operation, which suggested anastomotic leakage. The anastomotic stoma healed after washing by abdominal circulation through abdominal drainage tube. In this study, the positive rate of intraperitoneal contamination caused by ectopic bacteria NOSE was similar to that reported at home and abroad. Peng et al. (13) studied 30 cases of CRC patients undergoing laparoscopic lavage fluid culture after NOSE, of which 10 cases showed positive results, including 6 cases of E. coli, 1 case of *Enterococcus avium*, 1 case of gas-producing intestinal Bacillus, 1 case of *Klebsiella pneumonia*, and 1 case of *Enterobacter cloacae*; the positive rate was 33.3% (10/30). Ngu and Wong (14) performed laparoscopic NOSE surgery on 5 cases of CRC, used an incision during the operation protector step, collected and analyzed the peritoneal lavage fluid of 5 patients after operation, and found that one of them had a positive bacterial culture (*K. pneumonia* and *E. coli*, both sensitive to amoxicillin/clavulanic acid). The abdominal infection did not appear in any patient. Senft et al. (15) performed a NOSE operation on 12 German white pigs. The positive rate of postoperative peritoneal lavage fluid test was 58.3% (7/12); after the 14th day, the laparoscopic examination was performed again on the upper peritoneum and the lower peritoneum, and a pelvic swab culture was performed. The total number of contaminated peritoneal swabs was 33.3% (12/36), the bacterial load was very low, and there were no infectious complications. There was no difference in the peritoneal contamination rate compared with conventional laparoscopic-assisted surgery. However, Jonas D Senft and others reported that the rate of intraperitoneal contamination after NOSE was high. This may be due to the small total sample size, the anatomical factors of the experimental animals themselves, and the study mainly to assess the inflammatory response. Therefore, this study may be insufficient in the analysis of peritoneal contamination, but the report shows that the difference was not statistically significant. At the same time, Costantino et al. (16) reported that the contamination rate of peritoneal lavage fluid after NOSE was 100% and that in the non-NOSE group was 88.9%, P = 0.23. In general, the increase in the rate of contamination of the abdominal cavity by the NOSE procedure is higher than that of conventional laparoscopic-assisted surgery; however, statistically speaking, the rates of contamination of the two abdominal cavities are similar, and there is no significant difference. In this study, although some of the abdominal lavage fluid in the two groups was positive by bacterial culture, they did not continue to develop or cause local abdominal infections or even primary systemic infections. Therefore, the colorectal NOSE operation does not increase the chance of abdominal infection caused by ectopic bacterial contamination of the abdominal cavity due to the particular technique used in this operation.

Tumor cell shedding, ectopic implantation and postoperative complications, especially anastomotic leakage and pelvic and abdominal infections, have also been issues of concern to surgeons. Regarding the tumor-free principle of NOSE surgery, we evaluated it by collecting peritoneal wash fluid from patients for EC. In this study, by comparing the *EC* outcomes of peritoneal wash fluid exfoliation in the La-NOSE group and the CLA group, it was found that no tumor cells were found in the peritoneal washing liquid exfoliation cytology of all patients, and the positive detection rate was 0. Peng et al. (13) reported that 30 patients underwent peritoneal washing liquid cytology tests after NOSE. The results of bacterial culture were all negative, and the positive rate was also 0. Studies have shown that the positive rate of cytological detection of peritoneal washing liquid after radical resection of CRC is positively correlated with colorectal tumor stage (17, 18). This is a reasonable explanation for the fact that 50 patients undergoing laparoscopic-NOSE CRC radical surgery in this experiment did not detect tumor cells after exfoliation cytology of peritoneal washing liquid and did not improve ectopic tumor implantation, which met the principle of being tumor-free.

For the NOSE technique, the team strictly adheres to the principles of oncology, including high ligation of the mesenteric artery, lymph node dissection, and the scope of specimen resection. The operation of the entire surgical procedure is a high standard. In addition, the entire specimen extraction process of the team was carried out under the protection of a disposable sterile protective sleeve, and the abdominal cavity was repeatedly washed during the operation to avoid infection of the incision and implantation of the extraction site or pelvic tumor. Therefore, there are no additional technical obstacles that prevent oncology principles from being followed. Our results show that the average number of lymph node dissections in the La-NOSE group was 19.58, which exceeded the acceptable number of dissections, namely, 12 lymph nodes. Regarding postoperative anastomotic-related complications, especially anastomotic leakage and anastomotic stricture obstruction, their incidence rates (1/50 vs 2/75, *p=1.000*), as well as the rates of incision-related complications (0/50 vs 2/75, *p=0.516*) and other complications, were not significantly different between the two groups (*p>0.05*).

The present study confirmed that La-NOSES and CLA were similar to Ouyan’s study (19) in terms of elevated postoperative inflammatory indicators, with a corresponding increase in postoperative CRP and PCT, and the present study further indicated that both indicators were significantly elevated at POD2 and gradually recovered at POD7. Among them, La-NOSES caused a more significant and statistically significant increase in CRP and PCT. Similarly, there was some variability in the immunological indexes CD3 and CD4/CD8 specifically evaluated in this study. The elevation of CD3 in the La-NOSES group at POD2 and the recovery period at POD7 were statistically significant relative to for the CLA group. The response of CD4/CD8 in La-NOSES and CLA was elevated significantly at POD2 and was statistically significant.

The problems of NOSE in terms of sterile and tumor-free surgery and postoperative complications are worthy of close attention by surgeons. How can the negative impact of NOSE surgery on patients be reduced or even avoided? The combined experience of the surgical team suggested the following (1). Select an appropriate patient. Based on the patient’s preoperative examination, the tumor diameter should be less than 5 cm, the depth of infiltration should be below the serous layer, and patient’s BMI should be less than 28 kg/m^2^ (2). There should be a preoperative dietary adjustment, in which the patient eats easily digestible food with less residue 2-3 days before surgery (3). There should be prophylactic use of oral quinolones and metronidazole antibiotics before surgery, although the NCCN guidelines do not explicitly require it, and sufficient bowel preparation before surgery (4). Skilled surgeons should perform the laparoscopic operation techniques and maintain strict intraoperative aseptic operation criteria (5). There should be full washing with iodophor solution before opening the residual cavity (6). Protective sleeve use; the protective sleeve should be inserted into the abdominal cavity through the piercing hole, and the protective sleeve should be pulled out of the abdominal cavity through the residual cavity to form a similar intussusception structure (7). Before the specimen is removed, the protective sleeve should be placed into the anastomotic anvil (8). The specimen should be completely inserted into the protective sleeve, and the abdominal cavity of the protective sleeve should be tightened and then slowly removed. The anvil head should then be inserted into the proximal intestinal tube by the reverse puncture (9). Standardize and repeated peritoneal washing should be performed (10). There should also be useful postsurgical care measures and reasonable anti-infective treatment after surgery. This can reduce a series of problems caused by the unique NOSE operation technique.

This study also has limitations. First, this study is a non-complete randomized controlled study with a small sample size, which leads to some limitations in the results, and the sample size needs to be further expanded. Second, we did not evaluate the safety of the transvaginal route, considering that the transvaginal route would cause secondary damage and involve ethical issues. Third, this study reveals the early safety of NOSE, and the late safety has not been studied; thus, the 5-year follow-up time needs to be extended to understand its long-term outcome.

## Conclusion

Although the operation of the NOSE surgery is unique, the probability of ectopic bacterial contamination caused by the abdominal cavity is similar to that of CLA, and also does not cause ectopic implantation of tumor cells into the abdominal cavity. Inflammatory and immune indicators and postoperative complications also indicates that the early safety of NOSE surgery in the treatment of colorectal cancer is undoubtedly commendable, but its long-term results still need to be explored.

## Data Availability Statement

The raw data supporting the conclusions of this article will be made available by the authors, without undue reservation.

## Ethics Statement

The studies involving human participants were reviewed and approved by Ethics Committee of The First Affiliated Hospital of Wannan Medical College. The patients/participants provided their written informed consent to participate in this study.

## Author Contributions

Conception and design: LS, ZW, G L. Administrative support: LS and ZW. Provision of study materials or patients: LS, ZW, and GL. Collection and assembly of data: GL. Data analysis and interpretation: GL. All authors read and approved the final manuscript.

## Funding

This work was supported by the Scientific research project Anhui Provincial Health Commission in 2021(No.AHWJ2021b109).

## Conflict of Interest

The authors declare that the research was conducted in the absence of any commercial or financial relationships that could be construed as a potential conflict of interest.

## Publisher’s Note

All claims expressed in this article are solely those of the authors and do not necessarily represent those of their affiliated organizations, or those of the publisher, the editors and the reviewers. Any product that may be evaluated in this article, or claim that may be made by its manufacturer, is not guaranteed or endorsed by the publisher.

## Abbreviations

NOSE, natural orifice specimen extraction; CRC, colorectal cancer; EC, exfoliation cytology; CLA, conventional laparoscopic-assisted radical resection for CRC; La-NOSE, laparoscopic-assisted radical resection for CRC with NOSE; NCCN, the National Comprehensive Cancer Network.
